# Variation in Nectar Volume and Sugar Concentration of *Allium ursinum* L. ssp. *ucrainicum* in Three Habitats

**DOI:** 10.1100/2012/138579

**Published:** 2012-04-24

**Authors:** Ágnes Farkas, Réka Molnár, Tamás Morschhauser, István Hahn

**Affiliations:** ^1^Department of Pharmacognosy, Medical School, University of Pécs, Rókus u. 2. 7624 Pécs, Hungary; ^2^Department of Plant Systematics and Geobotany, Faculty of Natural Sciences, University of Pécs, Ifjúság u. 6., 7624 Pécs, Hungary; ^3^Department of Plant Taxonomy and Ecology, Loránd Eötvös University, Pázmány stny. 1., 1117 Budapest, Hungary

## Abstract

Floral nectar volume and concentration of ramson (*Allium ursinum* L. ssp. *ucrainicum*) were investigated in three different habitats, including two types of sessile oak-hornbeam association on brown forest soil with clay illuviation and a silver lime-flowering ash rock forest association on rendzina. Daily nectar production ranged from 0.1 to 3.8 **μ**L per flower with sugar concentrations of 25 to 50%. Mean nectar volumes and concentrations showed significant differences between freely exposed flowers and covered flowers, which had been isolated from flower visitors 24 h prior to nectar studies. Both the amount and quality of nectar were affected by microclimatic conditions and soil properties and varied between populations at different habitats. In the silver lime-flowering ash rock-forest association mean nectar volumes and concentrations were lower than in a typical sessile oak-hornbeam association on three occasions, the difference being significant in two cases. During full bloom, the date of sampling did not have a profound effect on either nectar volume or concentration.

## 1. Introduction

*Allium ursinum* L. (ramson or wild garlic) is a perennial plant, widely distributed in Europe, occurring in various deciduous woodlands, preferring damp shadow places, meso- and eutrophic, neutral to moderately acid soils of the hilly and the mountainous vegetation belt [1]. In Hungary, the largest populations can be found in Bakony and Mecsek hills, in the form of a continuous underwood layer in hornbeam-oak and beech forests [2, 3]. The flower stalk of ssp. *ursinum *is densely papillated and rough as opposed to the smooth pedicels of ssp. *ucrainicum *that lack papillae. The European distribution of ssp. *ursinum* is confined to the western and southern parts, being a subatlantic-submediterranean flora element, while ssp. *ucrainicum *is distributed in East Europe, with a western pontic-western sarmatic character [3]. The populations selected for the purposes of the present study belong to ssp. *ucrainicum*.

Besides being consumed fresh or cooked, ramson is a popular medicinal plant, lowering blood pressure, being effective against arteriosclerosis, diarrhea, and indigestion [4]. The plant is valued by bee keepers, as well, since ramson flowers can serve as pollen and nectar sources for honeybees, completing the spring bee pasture [5]. Ramson blooming starts in the second half of April and finishes in the first half of May. The umbel-like inflorescence comprises 8–12 trimeric flowers, with a septal nectary between the base of the ovary and the stamens of the inner circle, characteristic for the Alliaceae family [6–9].

 In the genus *Allium, *nectar secretion starts at the time of anthesis and ceases parallel with the wilting of the tepals, stamens, and style [10]. *Allium *species tend to secrete highly concentrated nectar: Akopyan [11] measured 70–75% sugar concentration in the nectar of *A. cepa*, while Hagler et al. [12] reported 52–65% for the same species. Kumar and Kumar Gupta [13] found similarly high concentrations in vegetable alliums, measuring 52.8–82.6% and 42.0–72.8% nectar sugar content in *A. cepa *and *A. fistulosum*, respectively. The 24 h sugar value in the latter two species varied between 0.219 and 0.767 mg/flower.

According to Silva et al. [14] nectar sugar concentration in *A. cepa* did not change significantly throughout the day, while mid- to late-morning and late evening peaks were observed in nectar volume. Rate of nectar secretion was influenced by both floral age and environmental factors, from which relative humidity was the most important, being significantly and inversely related to nectar production. Similarly, environmental factors were found to affect the nectar production of ramson, ranging from 0.16 to 0.42 mg nectar/flower/day, with an average of 52.13% sugar content [5]. In this study, sugar value was 0.14–0.25 mg in sunny weather, but remained below 0.1 mg in changeable, cool weather.

 Although the rewards offered by *A. ursinum *flowers can play an important role in the strengthening of bee colonies before the bloom of black locust (*Robinia pseudoacacia *L.), which is a major bee pasture in several countries, to date little is known about the nectar secretion process and nectar composition of ramson. Investigating the nectar traits of wild garlic can provide valuable information for beekeepers as well as for consumers of the honey derived from the floral nectar of *A. ursinum*. Although some data are available regarding the effect of environmental factors such as relative humidity and air temperature on nectar production in the* Allium* genus, the impact of different habitats on the nectar producing capacity of wild populations has largely been neglected. Therefore, the present study aims at demonstrating variation in nectar volume and sugar concentration in various populations of *A. ursinum* and at determining the possible role of habitat differences in this variation.

## 2. Materials and Methods

### 2.1. Location and Time of Studies

Field studies were done at three different locations in the Mecsek hills (South Transdanubia, Hungary) in the springs of 2007, 2008, and 2010. The selected sampling sites included two of the most dominant wood types and an edaphic one (for details see Tables 1 and 2).

### 2.2. 24-Hour Nectar Production Studies

Nectar was extracted with glass capillaries from 30 to 50 pollen-shedding flowers each day, at the time of peak nectar secretion, which was found to occur either at 9 hr or 17 hr in our pilot study. Each sampled flower represented a separate individual. In certain experimental designs the flowers have previously been isolated with a tulle net in order to exclude visiting insects (covered flowers). The volume of nectar produced in the preceding 24 hours was determined directly upon sampling the flowers with calibrated 5 *μ*L micro pipettes (DURAN), by reading the length of the nectar column within the capillary. The refractive index—corresponding to the concentration of nectar—was measured immediately with hand refractometers (ATAGO N-50E and OG 101/A). Since sucrose refractometers are calibrated directly in g sucrose per 100 g solution (% Brix) and the presence of hexose sugars scarcely affects the relationship between solute concentration and refractometer reading [15], the refractive index was directly used for characterizing the concentration of nectar.

 In addition, at site 3, repeated nectar sampling was performed from previously covered, pollen-shedding flowers on 5 consecutive days (15–19 April, 2007). All 5 study days fell within the main bloom of ramson. Each day, 25 to 30 flowers were sampled. Each flower was sampled only once during this period, that is, nectar was measured in different flowers on different days.

### 2.3. Statistical Analysis

Means of data measured in covered/uncovered flowers, at different sites and on different days were compared with either two-sample *t*-test or ANOVA with Tukey's multiple comparisons test. Homogeneity of variances was tested with *F*-test or Bartlett's test. If the variances differed significantly, Welch test was applied. The normality of data series was checked by using Kolmogorov-Smirnov test. If the normality assumption was violated, either Mann-Whitney test or Kruskal-Wallis test with Dunn's multiple comparisons post test was applied. For statistical evaluation of the results, the software GraphPad InStat (release 3.0.5) was used.

## 3. Results

### 3.1. The Effect of Flower Isolation on Nectar Volume and Concentration

Ramson flowers produced low to medium volumes (extreme values: 0.1–3.8 *μ*L/flower) of highly concentrated (extreme values: 25–55%) nectar at all three sampling sites on all occasions, with sugar values varying between 0.17 and 0.69 mg/flower in the three years of our study. The 24 h sugar values were within the range (0.219 to 0.767 mg/flower) calculated for the flowers of *A. cepa *and *A. fistulosum* [13], but were higher than the values determined in a previous study on *A. ursinum* (0.14–0.25 mg) [5].

The effect of 24-hour isolation of flowers preceding nectar measurements was investigated at site 3 on two different occasions (covered versus uncovered flowers in Table 2). In both cases, mean nectar volumes in covered flowers were significantly higher than in uncovered flowers (Table 3). Similarly, mean nectar concentration values of covered flowers exceeded those of freely exposed flowers in both years, but in 2010 the difference was not statistically significant (Table 4). The above results were taken into account in further evaluation of data, that is, data from covered and uncovered flowers were not pooled, and comparisons between various sites or dates were done either for covered flowers or freely exposed flowers.

### 3.2. Effect of the Habitat on Nectar Volume and Concentration

In order to analyze the effect of the habitat on nectar volume and concentration, ramson flowers that had not been previously isolated were sampled on three occasions. On April 27 mean nectar volumes differed significantly in 2007, but in 2008 we did not find any statistically relevant differences between the three study sites (Table 5). On 9 May, 2008 there was a significant difference in the mean nectar volumes of site 1 and site 2, and mean values at site 3 differed from those at the other two sites. Mean nectar volumes at site 2 were lower than at site 1 on all three days of investigation, the difference being significant in two cases.

Similarly to the amount of nectar, its mean concentration also showed significant differences at the three different habitats on both April 27, 2007 and May 9, 2008, but no such differences were found on April 27, 2008. Mean nectar concentrations were lower at site 2 on all three sampling dates compared to those measured at site 1—the difference being significant in two out of three cases (Table 6).

### 3.3. Effect of the Sampling Dates on Nectar Production

**Figure 1:**
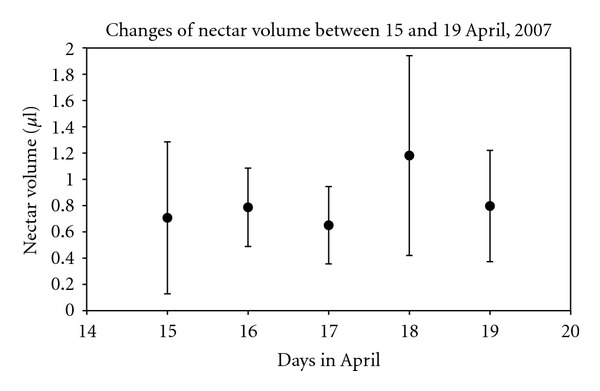
Nectar volume (mean and standard deviation) in covered ramson flowers at site 3, on five consecutive days of full bloom in April 2007.

**Figure 2:**
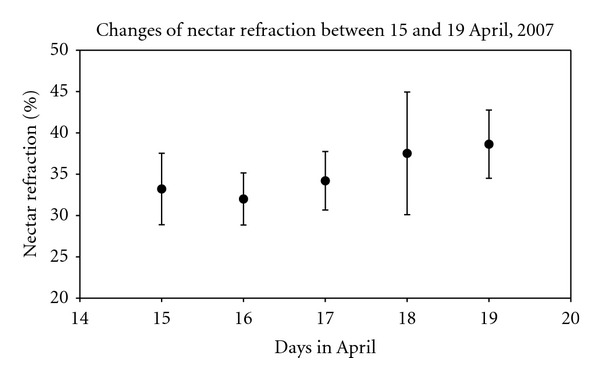
Nectar concentration (mean and standard deviation) in covered ramson flowers at site 3, on five consecutive days of full bloom in April 2007.

In 2007, previously isolated flowers were sampled on five consecutive days during full bloom at site 3. Neither nectar volume (Figure 1) nor concentration (Figure 2) changed significantly during this period.

## 4. Discussion

According to our previous studies, the nectar producing period lasts for 4 days in individual ramson flowers, with peak production on the 2nd day of anthesis [16]. This was in contrast with the study of Zimmermann and Pyke [17], who found that individual flowers of another mass-flowering species,* Polemonium foliosissimum*, produce equivalent nectar volumes every day of their lives within a single blooming season. Although the intensity of nectar production in *A. ursinum *flowers was expected to vary also at the population level on different days of full bloom, no significant differences were found either in volumes or concentrations of nectar on five consecutive days during full bloom. This might be explained by the different approach applied in the two studies: our previous investigation [16] monitored nectar secretion from the bud stage until flower senescence, sampling the same flowers on each consecutive day; whereas in the present study all flowers were at the stage of anthesis, and they were sampled on a single occasion.

Standing crop, that is, the quantity of nectar found in freely exposed flowers at a given time [15] tends to be lower than nectar volumes measured in isolated flowers, as demonstrated by several studies (e.g., [18]). The significantly higher nectar volumes of covered versus uncovered ramson flowers might be explained by the foraging activity of pollinators from freely exposed flowers. Various bees, including *Apis mellifera *L., *A. cerana *F., *A. dorsata *F., *A. florea* F. and *Trigona iridipennis* Smith, and flies like *Musca domestica *L., *Calliphora vicina *Robineau-Desvoidy, *Episyrphus balteatus *De Geer, *Eristalinus aeneus* Scopoli, and *Eupeodes *sp. have been reported as frequent visitors of *Allium *species [19–23]. In our field studies, the most important visitors of wild garlic flowers were honeybees and ants. The highly concentrated nectar reported for various *Allium *species [10–13] can make it difficult for honeybees to collect the secretion product. In our experience, ramson flowers might also produce nectar with concentration values exceeding 50%; however, the average values are in the range of 25 to 40%, which is suitable for honeybees, allowing even the production of unifloral wild garlic honey. Besides foragers, the slightly changed microclimate due to the coverage of inflorescences, which results in higher temperature and humidity, can contribute to differences in nectar production between covered and freely exposed flowers.

Differences in microclimate can also lead to variation observed between populations at different habitats. The rather diluted nectar in covered flowers at site 1 can be explained by the more humid microclimate in the closed oak-hornbeam association mixed with beech. The drier microclimate at the border of the sessile oak-hornbeam and sessile oak-Turkey oak woods in site 3 may stand in the background of large amounts of concentrated nectar even in isolated flowers. Interpopulational differences in nectar production were found in other plant species, as well: for example, in *Impatiens capensis* the variation in nectar volume was not significant among plants, but was nearly significant among populations [24]. Microclimatic conditions were found to influence nectar production in other melliferous plants like *Ajuga reptans*, *Lamium maculatum*, *Lamiastrum galeobdolon,* and *Ocimum basilicum* [25, 26]. For the latter species, physico-chemical soil properties were also found to be decisive: from the three investigated soil types, the highest intensity in nectar secretion was recorded on eutric cambisol, and daily nectar peaks were measured at various times depending on soil type: at 8 am on eutric cambisol, and at 10 am on fluvisol and humoglay [26].

 In our study, the humus content of the investigated soil types can be considered as good on luvisol (site 1 and 3) and excellent on leptosol (site 2, see Table 1), in accordance with the meso- and eutrophic soils preferred by ramson [1]. Ramson is known to prefer moderately acid soils, with the pH (H_2_O) ranging from 5.5 to 7.9 [27] or even from 6.0 to 7.5 [28], which corresponds to the values measured at site 2 (pH H_2_O 6.4). On the other hand, the pH values measured at the other two study sites fell below the optimal level. The relatively low pH values at site 3 may be responsible for the scattered appearance of ramson at this habitat, as opposed to the continuous coverage of ramson at the other two sites. The production of new roots was found to be inhibited by the even lower pH 3.6 in an experiment of Falkengren-Grerup and Tyler [29]. Low pH combined with high aluminium concentration has been reported to suppress root extension and biomass production [30].

Plants with different life histories and reproductive strategies (e.g., annuals versus perennials) may react differently to the availability of resources. Burkle and Irwin [31] demonstrated that nutrient addition increased aboveground biomass and flower production as well as nectar production in the monocarpic perennial *Ipomopsis aggregata *in the year of treatment; whereas in the perennial *Linum lewisii* reproductive output was not influenced by fertilization in the first year, but delayed effects were seen in the next year. The nectar secretion rate of *Vaccinium macrocarpon *was unaffected by fertilizer application [32]. Species-specific responses of nectar traits to variation in soil nitrogen availability were observed also by Baude et al. [33], who found that litter amendment to the soil led to an increase in total nectar sugar content in *Lamium amplexicaule*, but not in two other temperate grassland species, *Mimulus guttatus *and *Medicago sativa*. Besides sugar content, amino acid levels of the nectar can also be affected by soil conditions. Total amino acid concentrations varied significantly at both the plant and population level in *Impatiens capensis *[24]. In *Agrostemma githago,* total amino acid concentrations increased significantly with increasing fertilizer treatment [34].

*A. ursinum* applies Clan-of-Clone strategy which can be characterized among other things with relatively small allocation to vegetative reproduction, which prolongs local persistence [35]. Despite being a clonal plant, sexual reproduction is prevalent over clonal reproduction in the majority of natural populations [27, 28, 36]. Accordingly, *A. ursinum* can be characterized with extraordinarily high values of reproductive allocation, compared both to other woodland perennials and related species of the Liliales [37]. In a habitat that cannot provide enough nutrients during the time of flowering, the plant is not able to invest sufficiently into nectar production. This was demonstrated by our measurements as well. From the three study sites, Tubes (site 2) was the driest and warmest habitat, whose rendzina soil was characterized by the highest humus content and pH values. The high humus content can be advantageous if there is enough precipitation in spring—typically in April, at full bloom of ramson—since in this case nutrients are available in high amounts. Furthermore, rendzina soil is welldrained, which is important for ramson. Later on—typically in May, at the end of bloom—when there is less or no rain, the thin rendzina soil becomes warmer and drier, therefore humus decomposition is hindered and nutrients cannot be properly absorbed by ramson. This may account for the fact that nectar production was twice as high in April 2008 compared to May 2008 at site 2, as opposed to the less pronounced decrease in nectar production during the same period at site 1 (Table 5), characterized by a more humid microclimate and medium humus content. At site 3 the humus layer is rather shallow, and as ramson plants develop, the deeper penetrating roots reach a nutrient-poor soil layer, where lower levels of potassium, phosphorous, and nitrate-nitrogen can be measured [36]. The poorly drained soil with higher proportion of clay and the lack of sufficient nutrients may explain lower vigour of plants and consequently lower nectar sugar production.

## 5. Conclusion

Our study demonstrated that floral nectar volume and concentration varies in different populations of *A. ursinum*, which can be largely attributed to the varying conditions provided by different habitats. Populations in the sessile oak-hornbeam association, which is the typical habitat of ramson and provides sufficient nutrient levels for nectar secretion, produced higher volumes of nectar with higher nectar sugar concentrations, compared with the population in the silver lime-flowering ash rock forest, where *A. ursinum *cannot find its optimal living conditions.

## Figures and Tables

**Table 1:** Characteristics of the sampled forest stands.

Stand ID	Location; latitude (°); longitude (°); elevation (m); aspect; slope (°)	Bedrock; soil type; soil pH (H_2_O; KCl) H: humus content	Plant association; status; dominant species in canopy layer (c); shrub layer (s); herb layer (h)	Site description	Status of *Allium ursinum *ssp.* ucrainicum *
Site 1 Orfű valley	West-Mecsek hills N 46°07.041′; E 18°10.825′; 370 m; NE; 26°	Loess; brown forest soil with clay illuviation (luvisol); pH: 4.97; 4.05; H: 5.54%	Sessile oak-hornbeam association: *Asperulo taurinae-Carpinetum* Soó et Borhidi in Soó, 1962; zonal; c: *Carpinus betulus*, *Fagus sylvatica*, *Quercus dalechampii*; s: scarce; h:* Allium ursinum *ssp.* ucrainicum *	The middle of a typical occurrence of sessile oak-hornbeam forest.	Optimal, cool and humid; dominant

Site 2 Tubes hill	Mid-Mecsek hills; N 46°06.652′ ;E 18°11.899′; 535 m; S-SW; 26°	Limestone; rendzina soil (leptosol); pH: 6.37; 5.91; H: 6.93%	Silver lime-flowering ash rock forest association:* Aconito anthorae-Fraxinetum orni* (Borhidi-Kevey 1996); edaphic; c: *Tilia argentea*, *Quercus cerris, Q. pubescens *and *Q. virgiliana*, *Fraxinus ornus *s: *Cornus mas *h:* Allium ursinum *ssp.* ucrainicum *	Close to the border of the calciphilous oak association (*Tamo-Quercetum virgilianae*).	Not optimal, warm and dry; dominant

Site 3 Árpád peak	East-Mecsek hills N 46°08.511′; E 18°15.386′; 410 m; NE; 8°	Loess; brown forest soil with clay illuviation (luvisol);pH: 4.44; 3.51;H: 2.29%	Sessile oak-hornbeam association: *Asperulo taurinae-Carpinetum* Soó et Borhidi in Soó 1962; zonal; c: *Quercus dalechampii, Carpinus betulus*; s: sparse, *Crataegus oxyacantha, *h:* Melica uniflora, Allium ursinum *ssp.* ucrainicum *	Next to the border of Turkey oak wood. This habitat is receiving relatively more irradiation from the direction of the Turkey oak wood.	Not optimal, less humid, more acidic; mosaic appearance

**Table 2:** Sampling dates and sites, with bloom stage. C: covered flowers; UC: uncovered flowers.

Year	Date	Bloom stage	Site 1 Orfű valley	Site 2 Tubes hill	Site 3 Árpád peak
2007	April 14	Full	UC		UC
	April 15	Full			C
	April 16	Full			C
	April 17	Full			C
	April 18	Full	C		C
	April 19	Full			C
	April 26	End	C	UC	C
	April 27	End	UC	UC	
	April 28	End	C		

2008	April 25	Full			UC
	April 27	Full	UC	UC	C and UC
	April 29	Full	UC		C
	May 9	End	UC	UC	UC

2010	May 4	End			C and UC

**Table 3:** The effect of flower isolation on nectar volume at site 3.

	April 27, 2008			May 4, 2010		
	*n*	mean (*μ*L)	std	*n*	mean (*μ*L)	std
Covered	50	1.656*	0.930	32	0.637*	0.525
Uncovered	50	1.318*	0.677	32	0.172*	0.117

Method	Welch-test, *P* = 0.0415			Mann-Whitney test, *P* < 0.0001		

Abbreviations: *n*: sample size; std: standard deviation;*indicates significant difference between covered and uncovered samples.

**Table 4:** The effect of flower isolation on nectar concentration at site 3.

	April 27, 2008			May 4, 2010		
	*n*	mean (%)	std	*n*	mean (%)	std
Covered	50	38.340^∗ ^	4.556	32	33.250	6.754
Uncovered	49	35.898*	4.793	23	31.130	2.668

Method	*t*-test, *P* = 0.0108			Welch test, *P* = 0.1149		

Abbreviations:  *n*: sample size; std: standard deviation; *indicates significant difference between covered and uncovered samples.

**Table 5:** The effect of habitat on nectar volume.

	27 April 2007, end of bloom			27 April 2008, full bloom			9 May 2008, end of bloom		
	*n*	mean (*μ*L)	std	*n*	mean (*μ*L)	std	*n*	mean (*μ*L)	std
Site 1	33	1.339*	0.549	50	1.516	0.807	50	1.162*	0.549
Site 2	31	0.936*	0.526	50	1.422	0.772	50	0.732*	0.568
Site 3				49	1.318	0.677	50	0.104*	0.185

method	*t*-test, *P* = 0.0039			ANOVA, *P* = 0.4298			Kruskal-Wallis test, *P* < 0.0001		

Abbreviations: *n* = sample size, std = standard deviation; *indicates significant differences between sites.

**Table 6:** The effect of habitat on nectar concentration.

	April 27, 2007, end of bloom			April 27, 2008, full bloom			May 9, 2008, end of bloom		
	*n*	mean (%)	std	*n*	mean (%)	std	*n*	mean (%)	std
Site 1	33	36.182*	3.860	50	37.280	4.895	50	44.040*	4.247
Site 2	31	32.516*	3.548	50	35.640	4.129	50	40.080^∗ ^	4.597
Site 3				49	35.898	0.685	14	32.429^∗ ^	3.005

Method	*t*-test, *P* = 0.0002			ANOVA, *P* = 0.1655			ANOVA, *P* < 0.001		

Abbreviations: *n*: sample size; std: standard deviation; *indicates significant differences between sites.
